# Persistent vocal learning in an aging open-ended learner reflected in neural FoxP2 expression

**DOI:** 10.1186/s12868-024-00879-8

**Published:** 2024-07-04

**Authors:** Bushra Moussaoui, Kennedy Ulmer, Marcelo Araya-Salas, Timothy F. Wright

**Affiliations:** 1 Department of Biology, New Mexico State University, Las Cruces, NM 88003 USA; 2 Centro de Investigación en Neurociencias & Escuela de Biología, Universidad de Costa Rica, San José, Costa Rica

**Keywords:** Budgerigar, Cognitive senescence, FoxP2, Parrots, Vocal learning, Vocal plasticity

## Abstract

**Background:**

Most vocal learning species exhibit an early critical period during which their vocal control neural circuitry facilitates the acquisition of new vocalizations. Some taxa, most notably humans and parrots, retain some degree of neurobehavioral plasticity throughout adulthood, but both the extent of this plasticity and the neurogenetic mechanisms underlying it remain unclear. Differential expression of the transcription factor FoxP2 in both songbird and parrot vocal control nuclei has been identified previously as a key pattern facilitating vocal learning. We hypothesize that the resilience of vocal learning to cognitive decline in open-ended learners will be reflected in an absence of age-related changes in neural FoxP2 expression. We tested this hypothesis in the budgerigar (*Melopsittacus undulatus*), a small gregarious parrot in which adults converge on shared call types in response to shifts in group membership. We formed novel flocks of 4 previously unfamiliar males belonging to the same age class, either “young adult” (6 mo − 1 year) or “older adult” (≥ 3 year), and then collected audio-recordings over a 20-day learning period to assess vocal learning ability. Following behavioral recording, immunohistochemistry was performed on collected neural tissue to measure FoxP2 protein expression in a parrot vocal learning center, the magnocellular nucleus of the medial striatum (MMSt), and its adjacent striatum.

**Results:**

Although older adults show lower vocal diversity (i.e. repertoire size) and higher absolute levels of FoxP2 in the MMSt than young adults, we find similarly persistent downregulation of FoxP2 and equivalent vocal plasticity and vocal convergence in the two age cohorts. No relationship between individual variation in vocal learning measures and FoxP2 expression was detected.

**Conclusions:**

We find neural evidence to support persistent vocal learning in the budgerigar, suggesting resilience to aging in the open-ended learning program of this species. The lack of a significant relationship between FoxP2 expression and individual variability in vocal learning performance suggests that other neurogenetic mechanisms could also regulate this complex behavior.

## Background

Aging is commonly associated with progressive deterioration in many aspects of organismal function, including cognitive domains such as learning and memory [1, 2]. One complex behavior at the nexus of these two cognitive processes and thus vulnerable to age-related senescence, is language proficiency [2]. Vocal production learning, in which individuals produce new vocalizations based on the encoding of an auditory template and sensorimotor experience, is a key substrate for language development [3]. While language itself is considered unique to humans, vocal production learning is found in a handful of distantly related mammalian and avian taxa, most notably in songbirds and parrots. These avian vocal learners share convergent forebrain pathways with humans that contain analogous cerebral circuits for the detection, production and learning of vocalizations, and similar gene expression patterns within these circuits, which are absent in non-vocal learning taxa [4]. Such anatomical and molecular similarities make these avian taxa valuable models for studying learned vocal communication [5, 6].

The first gene to be definitively linked to human speech and language was the transcription factor forkhead box P2 (abbreviated FOXP2 when referring to the human protein, FoxP2 when referring to the protein in non-human animals, and *FoxP2* when referring to the mRNA form) [7]. Individuals possessing a mutation in this gene suffer from impaired orofacial fine motor control and deficits in language processing, as well as abnormal morphology and dysfunction in brain regions related to speech motor control such as the basal ganglia and Broca’s area [8, 9]. Following this discovery, FoxP2 has been extensively studied in avian vocal learning pathways, wherein its differential expression has been found to facilitate vocal flexibility in a variety of developmental and social contexts [10–14]. Knockdown of *FoxP2* in juvenile male zebra finches (*Taeniopygia guttata*) results in inaccurate vocal imitation and thus has been shown to prevent proper song development [15]. Also in this species, studies have shown that *FoxP2* mRNA expression increases in the striatal vocal control nucleus Area X during the juvenile sensorimotor learning period when song is nearing adult “crystallized” song [10] and is downregulated in Area X, relative to the adjacent striatum, when adult males sing in the absence of a female, producing more variable song syllables—during what is thought to be vocal “practice”—compared to when singing is directed towards a potential mate [16].

This link between FoxP2 expression patterns and vocal learning is further supported by the persistent downregulation of this gene in a taxon capable of life-long vocal learning, the parrots [13, 14]. While the zebra finch, and many other songbirds, are closed-ended learners, in which the ability to produce new vocalizations is restricted to an early developmental critical period after which adult songs stabilize, [5] parrots are open-ended learners and in captivity will exhibit extraordinary vocal mimicry that appears to persist throughout their adult lives [17]. Adult male budgerigars (*Melopsittacus undulatus*) exhibit consistently low *FoxP2* mRNA and protein expression in the parrot analogue of Area X, the magnocellular nucleus of the medial striatum (MMSt), regardless of vocal state, relative to the surrounding ventral striatum-pallidum (VSP) [13, 14]. This differential expression pattern observed in parrots has been termed persistent downregulation in previous literature, since it is observed regardless of a bird’s dynamic vocal state [13]. This persistent low level MMSt FoxP2 expression is consistent with the ongoing vocal plasticity that has been commonly observed in previous experimental studies with captive budgerigars. In captivity, adult male budgerigars readily imitate the contact calls of female mates, [18] and adult budgerigars of both sexes rapidly converge on shared contact calls in response to joining new social groups via a combination of imitation, improvisation, and recombination of frequency modulation patterns [19–21]. Although the budgerigar warble song is also a learned vocalization, it is a non-stereotyped vocalization and thus is not well suited for robust assays of vocal learning [22]. In a recent study investigating whether aging budgerigars exhibit a decline in vocal learning ability, we found that many components of vocal learning were maintained in this open-ended learner [23]. While it is assumed that this apparent resilience to senescence in open-ended vocal learners would be reflected in a correspondingly persistent FoxP2 downregulation pattern in aging adults, this relationship remains unconfirmed. Although Whitney et al. [14] included an adult group in their study of FoxP2 expression in developing budgerigars, these birds were only identified as being greater than 120 days (i.e. adults of indeterminant age). To date, FoxP2 expression has not been characterized in different adult age groups of an open-ended vocal learner. Additionally, a correlation between behavioral vocal learning measures (such as the degree of acoustic similarity between social associates) and FoxP2 striatal expression has not been established.

In this study, we tested whether aging affects adult vocal learning and its neural underpinnings in an open-ended learner. To do this, we examined collected neural tissue from birds in our previously published study [23] in which we conducted a vocal learning assay of male budgerigars of two different adult ages (“young adult”: 6 mo – 1 year.; “older adult” ≥ 3 year.). Immunohistochemistry was performed to measure FoxP2 protein expression in our region of interest, the MMSt, and the surrounding striatum (VSP), as a reference region, to determine if expression profiles differ between young and older adults. We hypothesized that persistence of vocal learning in open-ended learners is related to patterns of FoxP2 expression characteristic of vocal flexibility. We predicted that older birds will not exhibit diminished vocal learning ability and thus will have a similarly low FoxP2 MMSt/VSP expression ratio compared to young adults. We also predicted that individual variation in vocal learning can be explained by FoxP2 MMSt/VSP expression such that individuals demonstrating greater vocal learning will exhibit lower expression of FoxP2.

## Methods

### Behavioral vocal learning assay

**Fig. 1:**
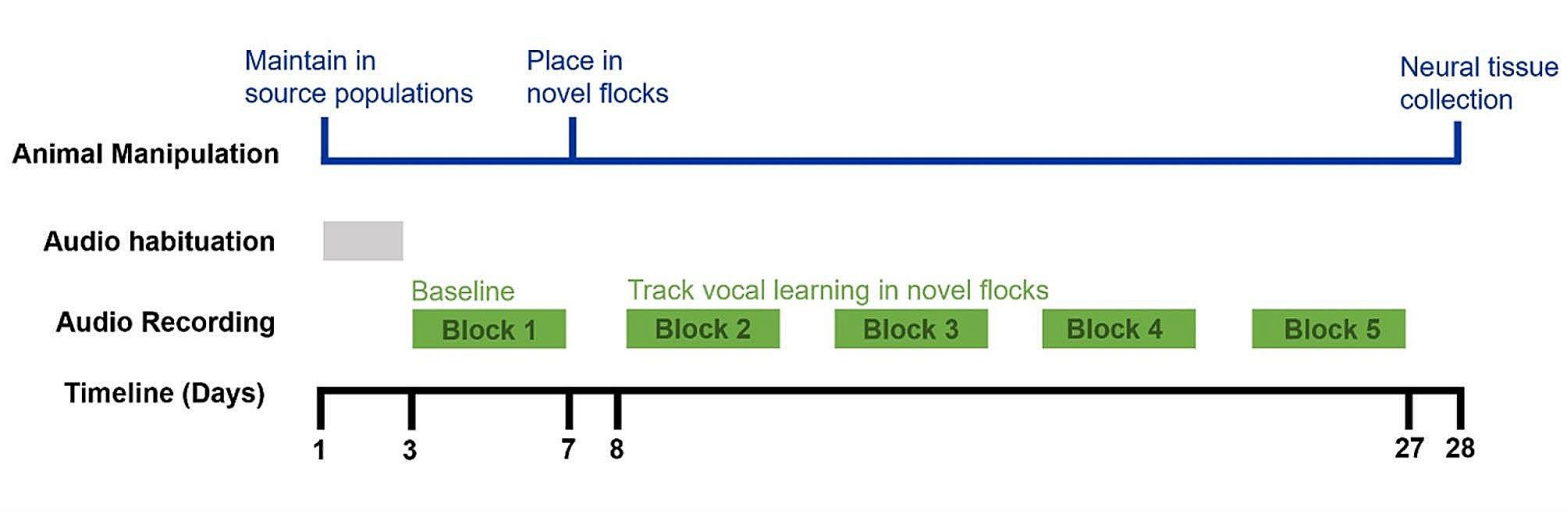
Experimental timeline. Experimental timeline outlining the formation of novel flocks, audio-recording of vocalizations, and neural tissue collection

To assess the effect of adult age on vocal learning ability, we formed novel flocks of adult male budgerigars (*Melopsittacus undulatus)*, belonging to the same age class, either “young adult” (6 mo – 1 year) or “older adult” (≥ 3 year) who were close to or exceeding the mean life expectancy of 4.57 years for this species in captivity, [24] and then collected audio-recordings from all individuals over a 20-day learning period to measure changes in contact call repertoires over time and assess age-related differences in call learning (Fig. 1). Full details of this behavioral vocal learning assay are described in Moussaoui et al., [23] as a subset of birds from that main experiment were used in this study focused on the neural underpinnings of age-related differences in vocal learning ability. In brief, 24 birds of each adult age class were acquired from a commercial breeder (McDonald Bird Farms, Kerrville, TX) and from our own research colony at the New Mexico State University Animal Care Facility, which was derived of birds from the same breeder. The commercial breeder provided young birds and old birds housed in four separately built aviaries and three separately built aviaries, respectively, from a total breeding population that exceeds 10,000 individuals and thus is unlikely to be inbred. These birds, in combination with a set of old males from our colony, generated four independent source populations for each age class such that birds originating from different populations were socially and acoustically unfamiliar and could thus be combined to form flocks of novel membership. Six replicate flocks were formed for each adult age class with each flock being composed of four individuals, such that each individual in a replicate flock originally belonged to a separate source population and thus was unfamiliar to its flockmates. Flocks were housed in 79 × 52 × 102 cm cages with matching layouts of perches, food dishes, and water. Prior to novel flock formation, baseline contact call repertoires were collected for each individual during a 4-day audio-recording block (block 1 in Fig. 1). Upon being placed with novel flockmates, birds were audio-recorded daily across four 4-day blocks (blocks 2–5 in Fig. 1). At the end of this vocal recording period, neural tissue was collected for a randomly selected subset of individuals to measure expression of a key vocal learning related gene. All procedures conducted in this study were approved by the New Mexico State University Animal Care and Use Committee (protocol number 2020-030).

### Acoustic analysis to measure vocal learning

Contact calls were isolated from these audio-recordings using a semi-automated signal detection procedure in the R package *ohun* (*version 1.0.0*) [25] in R version 4.0.5 [26]. This involved applying optimized amplitude, frequency, and duration thresholds, the use of supervised random forests to classify detections as “signal” (contact calls) or “noise” (other vocalization types, feather ruffling, cage rattling, background flockmates), and lastly a manual quality control step in which we visually confirmed detections classified as contact calls. Seventeen standard acoustic features were then measured from contact call spectrograms, including various features related to the distribution of power in the time and frequency domains, using the R package *warbleR* (*version 1.1.27*) [27]. The dimensionality of these multiple extracted acoustic measures was reduced using t-Distributed Stochastic Neighbor Embedding (t-SNE) [28] implemented in the R package *Rtsne* (*version 0.15*) [29]. We mapped contact calls in an acoustic trait space, hereafter “acoustic space”, generated by projecting the first two dimensions such that acoustically similar calls appear closer together in space. We then quantified the kernel density area of each individual’s acoustic space subset by recording block using the R package *PhenotypeSpace* (*version 0.1.0*) [30] to assess changes in contact call repertoires over time. All acoustic spaces were generated using the same number of contact calls (180) using a rarefaction subsampling procedure to get the mean area from 30 equal size randomly selected subsets.

From these acoustic space areas, three vocal learning measures were computed for each bird. Firstly, we defined *vocal diversity* as the change in acoustic space area of an individual’s contact call repertoire from the beginning of the vocal learning assay (audio-recording block 1) to the end (audio-recording block 5). Secondly, we defined *vocal plasticity* as 1 minus the intersection over union of an individual’s beginning and ending acoustic space areas, where higher values indicate less acoustic similarity between initial and final contact calls, and thus greater vocal plasticity. Thirdly, we defined *vocal convergence* as the intersection over union of an individual’s acoustic space area and the combined acoustic space area of its flockmates at the end of the vocal learning assay, where higher values indicate greater matching of ones’ contact call repertoire to that of its social group.

### Neural tissue collection and preparation

Following the vocal learning assay (Fig. 1), two birds from each flock of 4 birds (*N* = 12 individuals per age class) were randomly selected for sacrifice to collect whole brains for neural analysis of the *FoxP2* gene. As previous work has shown that adult budgerigars exhibit persistent downregulation of FoxP2 regardless of vocal state, we did not record vocal output or time spent vocalizing immediately prior to euthanasia [13]. On day 28 of the experimental timeline, selected birds were euthanized via an overdose inhalation of isoflurane and whole brains were extracted and flash frozen within 5 min using liquid nitrogen and then stored at -80 °C until later use. All collected brains were cryosectioned coronally using a Leica CM1850 cryostat (Leica Microsystems) at -20 °C. The left or right hemisphere of each brain was randomly selected for extraction of 1 mm deep punches with an 18 gauge Luer stub from both MMSt and VSP for future RNA isolation work. The non-punched hemisphere (young adults: *N* = 6 RH, 3 LH; older adults: *N* = 5 RH, *N* = 4 LH) was used in this study for immunohistochemical staining for FoxP2 protein expression. Sections of 20 μm thickness were thaw-mounted onto positively charged slides (Fisher Scientific) in 7 replicate series and stored at -80 °C. One series was Nissl stained for visualization of cytoarchitectural boundaries to enable identification of the key brain regions of interest, MMSt and its adjacent striatum. With reference to the budgerigar brain atlas, [31] adjacent slides were selected for immunohistochemical staining.

### Immunohistochemical staining for FoxP2

Brain sections were first fixed with 4% paraformaldehyde (titrated with NaOH and HCl to achieve a pH of 7) for 5 min, dip-rinsed twice with 1X phosphate buffered saline (PBS), then rinsed three times with 1X PBS with 0.4% Triton X-100 (PBST) for 5 min each. Slides were then blocked with 5% sheep serum (Sigma-Aldrich) in PBST for 1 h at room temperature to prevent nonspecific binding followed by overnight incubation at 4 °C in the FoxP2 primary antibody (Mouse, 1:500, Thermo Fisher Scientific) solution. Slides were then rinsed in 1X PBST three times at 5 min each prior to incubation in the Alexa Fluor 594 secondary antibody (Goat anti-mouse, 1:200, Thermo Fisher Scientific) for 2 h at room temperature. Sections were then rinsed four times at 5 min each in 1X PBS, once in ddH20, and finally coverslipped using Vectashield with 405 nm excitable DAPI (Vector Laboratories). Negative controls were performed identically as above except for the omission of primary antibody.

### Quantification of FoxP2 expression

**Fig. 2:**
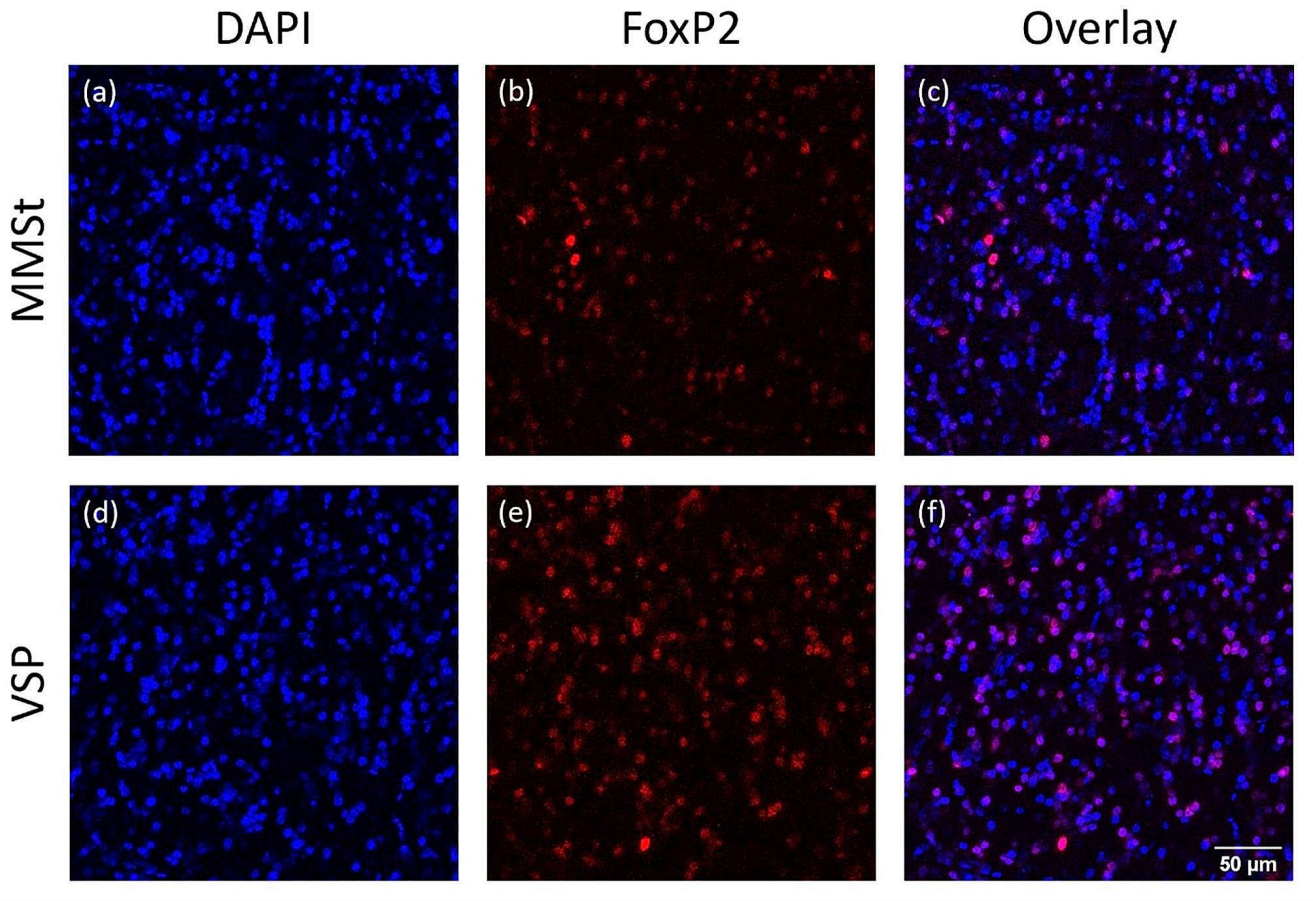
FoxP2 differential expression. Immunohistochemical staining of FoxP2 protein in the magnocellular nucleus of the medial striatum (MMSt) and the adjacent striatal non-vocal learning region (VSP). Images were taken at 40X magnification using a confocal microscope. These example images are from a single older adult male budgerigar. Blue signal (**a** and **d**) indicates DAPI stained cell nuclei, representing the total number of cells present in the area imaged. Red signal (**b** and **e**) indicates FoxP2 labeled cell nuclei. DAPI and FoxP2 labeled images are overlaid (**c** and **f**) to identify the percentage of total cells that express FoxP2

Following immunohistochemistry, tissue slides were imaged using a TCS SP5 II Confocal microscope (Leica Microsystems) to capture fluorescent images for quantification of FoxP2 protein expression. Images were taken within the MMSt and VSP regions from each of two sections per bird at 40X magnification. Images of each region were taken sequentially between frames for each channel (405 nm for DAPI, 594 nm for FoxP2, and their overlay) and saved as TIFF image files (Fig. 2). Images were imported into ImageJ 1.53e (NIH), [32] converted to an 8-bit grayscale and auto-thresholded. DAPI and FoxP2 labeled cells were then manually counted using the multi-point tool while referencing cell morphology in original images. To avoid counting noise arising from secondary antibody background staining, FoxP2 labeled cells were only counted if they overlaid atop a DAPI counted cell. For each image, FoxP2 cell counts were divided by the total number of cells (DAPI counted cells) yielding a percentage of neural cells that were expressing FoxP2 in the MMSt and the VSP, which was then used to calculate a MMSt/VSP FoxP2 expression ratio per section. This MMSt/VSP ratio was averaged for the two imaged sections per bird. Cells were counted by two trained observers and inter-observer reliability was assessed at “good” to “excellent” (ICC = 0.965; 95% CI = [0.769, 0.995]) by employing a single-measurement, absolute-agreement two-way mixed-effects model using the package *irr* (*version 0.84.1*) [33].

### Quantification and statistical analysis

All statistical analyses were carried out in R version 4.2.1 [26] using the *stats* (*version 4.2.1*), *car* (*version 3.1-1*), and *brms* (2.18.0) packages. Statistical significance was determined with an alpha level of 0.05 for frequentist analyses, or a 95% credible interval that did not include zero for Bayesian analyses. To test whether FoxP2 expression differs by adult age, we conducted independent samples t-tests for FoxP2 expression in MMSt, VSP, and the MMSt/VSP expression ratio. To determine whether neural FoxP2 expression predicts vocal learning, and whether this relationship differs between young and older adults, we fit Bayesian generalized linear models, with each of the three vocal learning measures (vocal diversity, vocal plasticity, and vocal convergence) as a response variable in three separate models and adult age class and mean centered FoxP2 MMSt/VSP expression as explanatory variables. Regressions were run in Stan [34] through the R package brms [35]. Response variables expressed as proportions (vocal plasticity and vocal convergence) were modeled with a Beta distribution while vocal diversity was modeled with a normal distribution. Effect sizes are presented as median posterior estimates and 95% credibility intervals as the highest posterior density interval. Minimally informative priors were used for population-level effects. Models were run on four chains for 10,000 iterations, following a warm-up of 10,000 iterations. The effective sample size was kept above 3000 for all parameters. Performance was checked visually by plotting the trace and distribution of posterior estimates for all chains. We also plotted the autocorrelation of successive sampled values to evaluate the independence of posterior samples, kept the potential scale reduction factor for model convergence below 1.01 for all parameter estimates and generated plots from posterior predictive samples to assess the adequacy of the models in describing the observed data.

Although, vocal learning measures were computed for each recording block, here we only examine vocal learning measures computed during the last audio-recording block, as this block was closest in time to neural tissue collection, and FoxP2 expression levels more accurately reflect more recent vocal behavior [11]. We extracted this vocal data for each of the 12 young adult and 12 older adult birds for which we had measured FoxP2 MMSt/VSP expression, the key measure that has been linked to persistent vocal learning ability [13, 14]. Four of the older adults, however, had produced fewer than 6 contact calls during the last audio-recording block (2 birds produced 4 calls, and 2 birds did not call), failing to meet our minimum threshold for accurate measurements and comparisons of acoustic space and thus could not be included in this analysis, leaving a sample size of 8 older adults for analyses of call learning.

## Results

### Similar FoxP2 protein expression in young and older adults

**Fig. 3:**
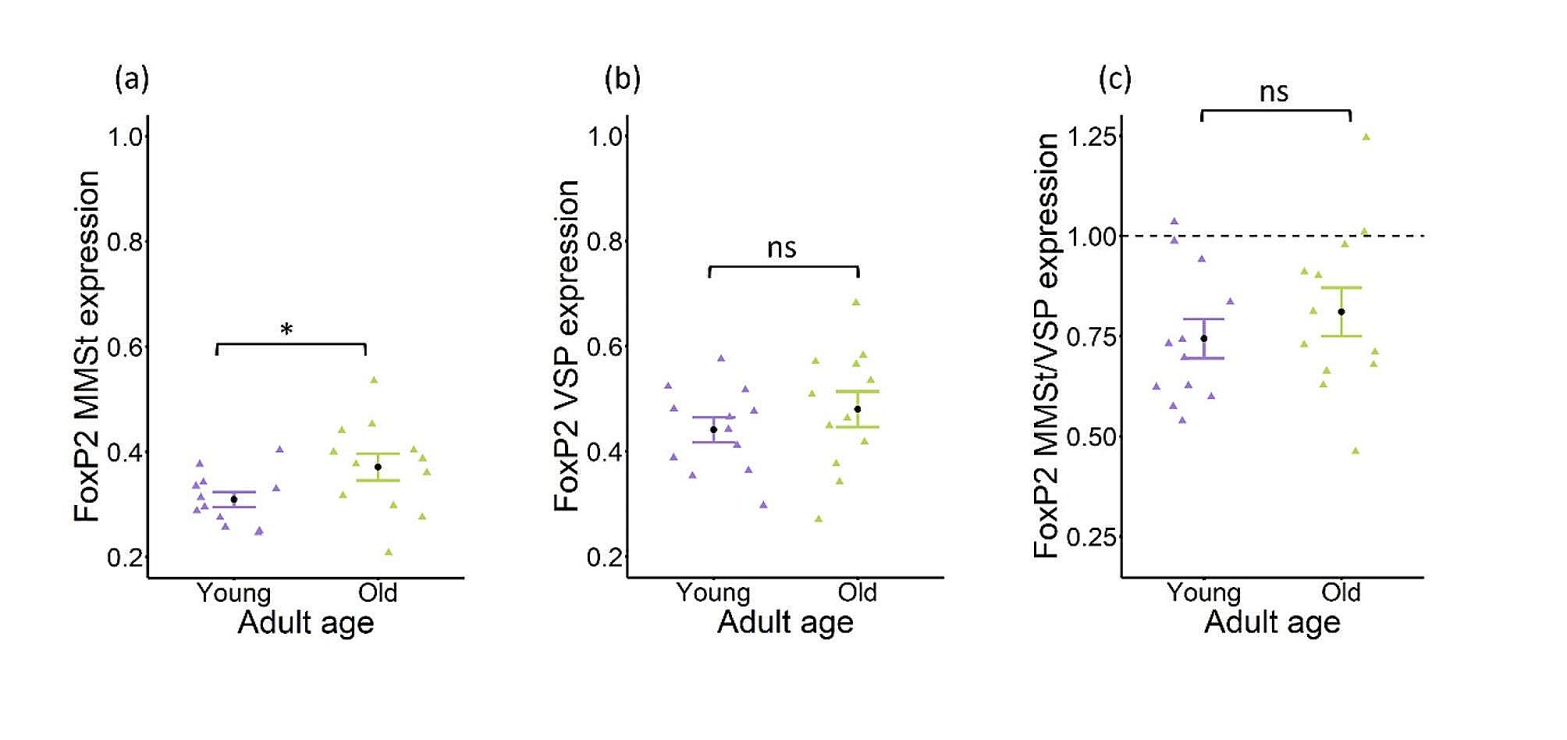
Low FoxP2 expression ratio maintained in older adults. Mean and standard error plots display FoxP2 protein expression levels in the striatal vocal learning nucleus, magnocellular nucleus of the medial striatum (MMSt), and the adjacent striatum in young (*N* = 12) and older adults (*N* = 12). (**a**) Proportion of MMSt cells expressing FoxP2. (**b**) Proportion of non-vocal learning adjacent striatum, VSP, cells expressing FoxP2. (**c**) Ratio of expression percentages (MMSt/VSP). A ratio of 1, as marked by the horizontal dashed line, indicates equal expression levels in MMSt and VSP. A ratio below 1 indicates lower expression of FoxP2 in the MMSt, relative to VSP, and a ratio above 1 indicates higher expression of FoxP2 in the MMSt, relative to VSP. Circles represent the mean, triangles represent individual expression values, and error bars represent the standard error. *P < 0.05; ns = not significant

Young adult budgerigars exhibited a significantly lower mean proportion of MMSt cells expressing FoxP2 compared to older adults (young adults = 0.309 ± 0.014 (SE), older adults = 0.371 ± 0.025 (SE), *t* = -2.12, *df* = 22, *p* = 0.045) but the two age classes did not significantly differ in either the mean proportion of non-vocal learning adjacent striatum (VSP) cells expressing FoxP2 (young adults = 0.441 ± 0.024 (SE), older adults = 0.480 ± 0.034 (SE), *t* = -0.95, *df* = 22, *p* = 0.35) or the mean MMSt/VSP expression ratio (young adults = 0.744 ± 0.049 (SE), older adults = 0.811 ± 0.061 (SE), *t* = -0.86, *df* = 22, *p* = 0.40) (Fig. 3a-c). Both young and older adult age classes budgerigars exhibited lower FoxP2 expression in the MMSt vocal learning nucleus compared to the non-vocal learning adjacent striatum, VSP (Fig. 3c). All individuals displayed low FoxP2 expression in MMSt except for two older adults who had MMSt/VSP expression ratios of 1.00 and 1.25 and one young adult with a ratio of 1.03.

### Vocal plasticity and vocal convergence maintained in older adults

**Fig. 4:**
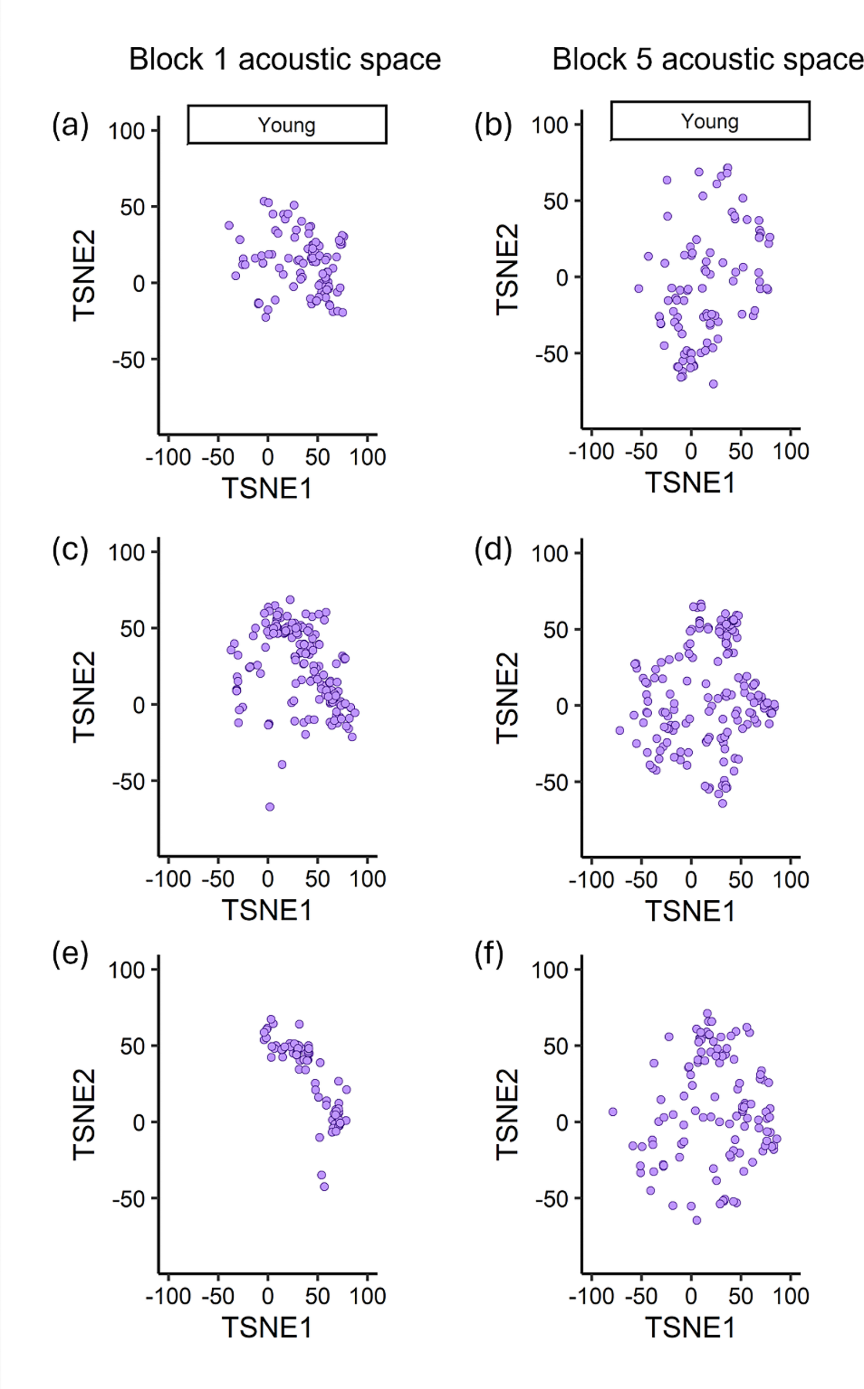
Sample of changes in young adult acoustic space. Acoustic trait space of 3 young adult budgerigars, where each point represents a single call characterized by seventeen standard acoustic features and projected into two-dimensional space using the t-SNE dimensionality reduction approach. Acoustically similar calls appear closer together in this trait space defined by the dimensions TSNE1 and TSNE2. Calls produced by individual 1 in recording block 1 (**a**) vs. block 5 (**b**), individual 2 in recording block 1 (**c**) vs. block 5 (**d**), and individual 3 in recording block 1 (**e**) vs. block 5 (**f**)

**Fig. 5:**
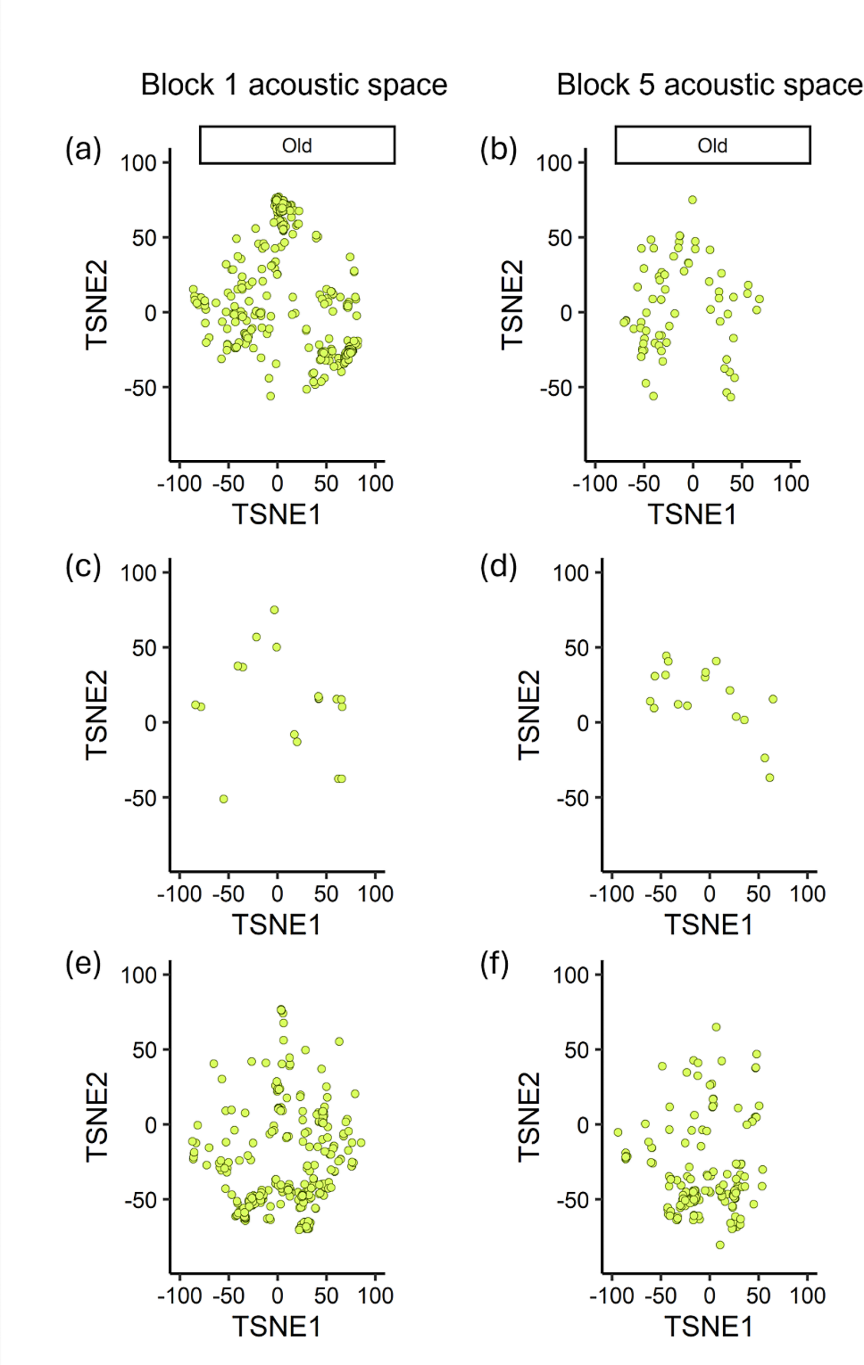
Sample of changes in older adult acoustic space. Acoustic trait space of 3 older adult budgerigars, where each point represents a single call characterized by seventeen standard acoustic features and projected into two-dimensional space using the t-SNE dimensionality reduction approach. Acoustically similar calls appear closer together in this trait space defined by the dimensions TSNE1 and TSNE2. Calls produced by individual 1 in recording block 1 (**a**) vs. block 5 (**b**), individual 2 in recording block 1 (**c**) vs. block 5 (**d**), and individual 3 in recording block 1 (**e**) vs. block 5 (**f**)

For our three learning measures, adult age was only found to significantly explain the difference in vocal diversity between an individual’s beginning and ending contact call repertoires (95% CI [-1.974, -0.172]) (Table 1). Young adults exhibited an increase in acoustic area, indicating an increase in vocal diversity, while older adults, exhibited a decrease in acoustic area and thus a loss in vocal diversity over the course of the three-week vocal learning assay (Figs. 4 and 5). Young and older adults did not significantly differ with respect to vocal plasticity or vocal convergence (Table 1).

**Table 1:** Adult age predicts vocal diversity. Results from Bayesian generalized linear models assessing the explanatory power of FoxP2 MMSt/VSP expression ratio (mean centered), adult age, and their interaction, in predicting each of the three vocal learning measures computed for audio-recording block 5. Negative estimates for effect sizes for Age indicate lower values for the older adult age class

			95% Credible Interval	
Response	Fixed effect	Estimate	Lower	Upper
Vocal diversity	FoxP2	-0.461	-3.672	2.717
	**Age**	**-1.071**	**-1.974**	**-0.172**
	FoxP2 x Age	-0.747	-5.444	3.979
Vocal plasticity	FoxP2	-1.564	-4.795	1.603
	Age	0.080	-0.816	0.969
	FoxP2 x Age	-0.901	-5.651	3.935
Vocal convergence	FoxP2	-0.506	-3.586	2.669
	Age	-0.677	-1.499	0.167
	FoxP2 x Age	1.079	-3.632	5.598

Bold font indicates a factor whose credible interval for estimated effect size does not include zero

### Individual variation in vocal learning ability not predicted by FoxP2 expression

**Fig. 6:**
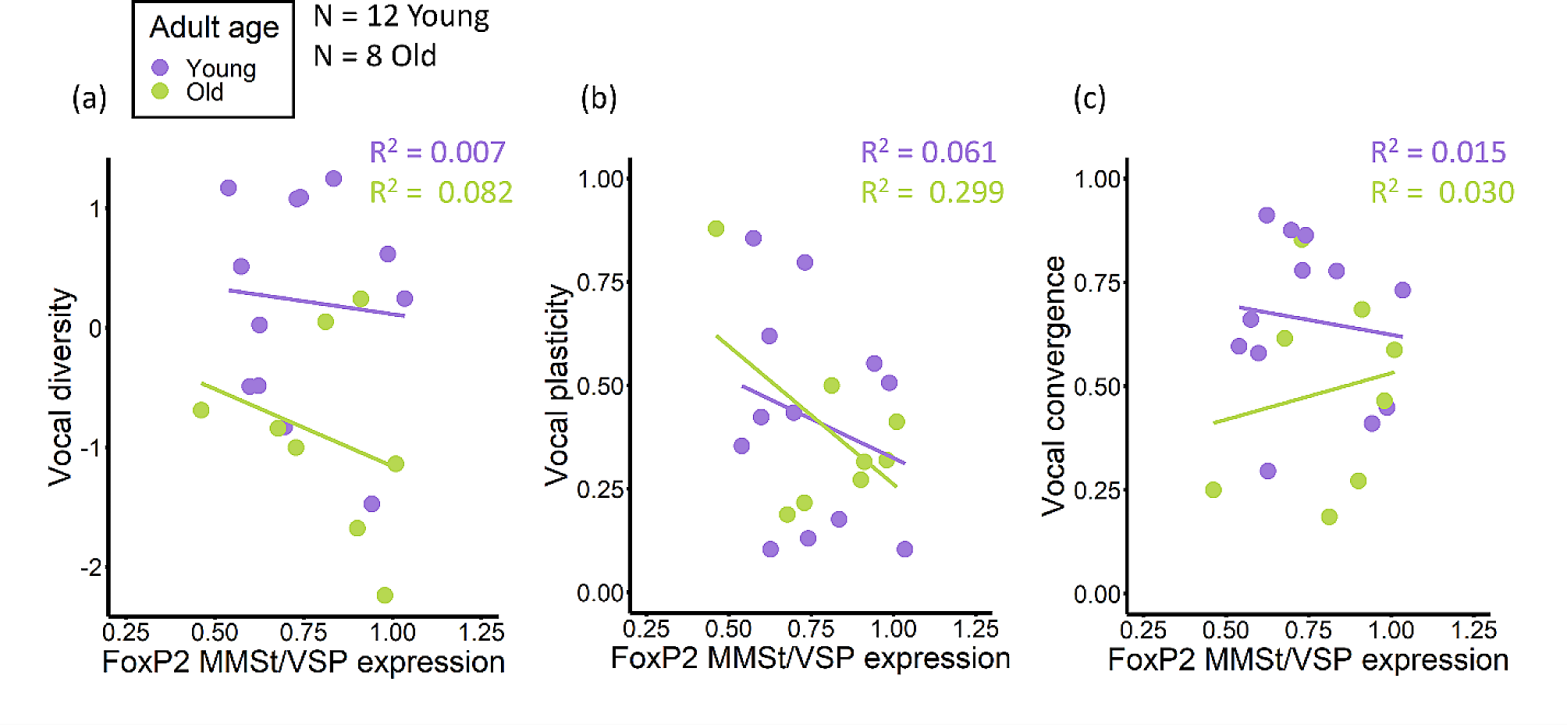
FoxP2 expression and individual variation in vocal learning. Scatterplots of the relationship between vocal learning measures and FoxP2 protein expression ratio (MMSt/VSP), fit linearly for each adult age class. FoxP2 expression ratios below 1 indicate lower FoxP2 expression in the vocal learning striatal nucleus, MMSt, relative to the adjacent striatum, VSP. (**a**) Change in the area of an individual’s acoustic space from the beginning of the vocal learning assay (audio-recording block 1) to the end (audio-recording block 5), where 0 indicates no change in vocal diversity, positive values indicate an increase in vocal diversity, and negative values indicate a decrease in vocal diversity. (**b**) 1 minus the intersection over union of an individual’s beginning and ending acoustic spaces, where higher values on the y-axis indicate greater vocal plasticity. (**c**) Intersection over union of an individual’s acoustic space and the combined acoustic space of its flockmates, where higher values indicate greater vocal convergence

Neural FoxP2 expression was not found to significantly explain individual variation in vocal learning ability (Fig. 6; Table 1). Some trends, however, support a role of low FoxP2 expression in MMSt as a facilitator of vocal learning. For instance, the negative relationship between vocal diversity and FoxP2 expression observed for both age classes, although non-significant, is consistent with birds that exhibited larger increases in acoustic area having lower FoxP2 expression in the MMSt (Fig. 6a). Additionally, the negative relationship between vocal plasticity and FoxP2 expression for both age classes, although non-significant, does match expectations of lower FoxP2 levels in individuals exhibiting higher vocal plasticity (Fig. 6b).

## Discussion

Differential expression of FoxP2 in vocal learning brain nuclei (Area X in songbirds and MMSt in parrots) relative to adjacent non-vocal learning regions is associated with both social context-dependent periods of vocal variability [16] as well as active periods of vocal learning [14]. A key factor differentiating taxa as closed-ended learners (vocal learning restricted to an early life sensitive period) or open-ended vocal learners (vocal learning continuing into adulthood) is thought to be the persistent downregulation of FoxP2 in the vocal learning nuclei of adult learners [13]. Studies of FoxP2 expression patterns in open-ended learners, however, have defined adults as a single age class, leaving it unclear whether this vocal learning-related FoxP2 expression pattern is similar for adults of different ages, as is generally assumed. In this study, we sought to better understand age-related changes in vocal flexibility in an open-ended learner by conducting a vocal learning assay in which we formed novel flocks of either young adult budgerigars or older adult budgerigars, tracked contact call production over time, and measured neural FoxP2 expression levels from a subset of individuals. Findings of generally similar neural FoxP2 expression and vocal learning ability between young and older adults suggest that these open-ended learners largely maintain vocal flexibility into later adulthood, with the exception of diminished vocal diversity.

### Persistent FoxP2 downregulation maintained in old age

Although older adult budgerigars exhibited a significantly higher percentage of MMSt cells expressing FoxP2 compared to young adults, they exhibited similarly low expression of FoxP2 in MMSt relative to the non-vocal learning adjacent striatum, VSP. This pattern of persistent FoxP2 downregulation, which is not observed in closed ended learners such as the zebra finch, has been identified by previous studies as a defining feature of open ended learning [13, 14]. Previous studies in adult budgerigars reported similar FoxP2 MMSt/VSP expression ratios as we found, although adults in these studies were broadly defined as being at least 120 days old, which is roughly at least 0.3 years old [13, 14]. Given that budgerigars have a mean life expectancy of 4.57 years, [24, 36] our findings of persistent downregulation in older adults in at least their 3rd year of age, suggest that neural plasticity in vocal learning circuits, as regulated by FoxP2, persists close to the end of the average life span of these open-ended learners. The older adult individual exhibiting the highest MMSt/VSP expression ratio of 1.25 was a bird obtained directly from our research colony and thus had a known hatch date of August 29, 2016 and could reliably be aged at 4.6 years old. Other older adults of comparable ages to this individual (and for whom hatch date is also known), ranging from 4.3 to 4.8 years old, had much lower expression ratios, ranging from 0.66 to 0.73, eliminating the possibility that the outlying high expression in this individual is due to a much older age than the rest of his cohort. Additionally, the lowest FoxP2 MMSt/VSP expression ratio, 0.46 was observed in an older adult, further supporting the maintenance of the neural underpinnings of vocal flexibility into old age in an open-ended learner.

We did find an age-related difference in absolute MMSt expression of FoxP2, with higher expression levels observed in older adults. This pattern is similar to previous findings of significantly higher absolute MMSt FoxP2 expression in adult budgerigars compared to juveniles and yet a similarly low FoxP2 MMSt/VSP expression ratio [14]. While previous studies have focused on persistent downregulation of FoxP2 as a defining characteristic of open-ended vocal learning, this finding suggests that the expression level of FoxP2 in MMSt may also explain the trajectory of vocal learning ability throughout the lifespan of an open-ended learner. Further investigation of this relationship would be valuable.

### Role of FoxP2 expression in facilitating vocal learning

Vocal learning measures were generally similar between age classes. While we did see a difference in vocal diversity (amount of acoustic space covered by an individual’s calls) between the two age classes, we saw no significant differences in vocal plasticity (acoustic dissimilarity between individuals’ starting and ending contact call repertoires) or vocal convergence (acoustic similarity between individuals’ contact calls to those of their flockmates at the end of the vocal learning assay). These findings generally support our hypothesis that older budgerigars are resilient to aging with respect to altering their calls and matching the calls of their flockmates. In our larger study, from which a subset of birds was randomly selected for this study, reduced vocal diversity in older birds coincided with fewer and weaker affiliative social bonds, thus it may be that this component of vocal learning is constrained more by social context than an age-related cognitive decline [23]. A previous study of age-related changes in song traits in female European starlings (*Sturnus vulgaris*), which are considered open-ended vocal learners, similarly reported a reduction of repertoire size in older adults in both cross-sectional and longitudinal analyses [37]. Other studies, however, have found the opposite trend, such as an investigation of age-dependent song variation in open-ended learning collared flycatchers (*Ficedula albicollis)*, which found that repertoire size increased with adult age as sampled from adult males with known ages ranging from 2 to 7 years old [38, 39].

Although young and older adults did not differ in two out of the three measures of vocal learning, we did expect individual vocal variation to be explained by individual variation in neural FoxP2 protein expression levels. MMSt/VSP expression ratios of this gene, however, were not found to significantly predict any vocal learning characteristic we measured. This result may be explained by our sample sizes not capturing a wide enough range of individual variability in each of these measures to establish such a relationship, should it exist. It is worth noting that the relationship between FoxP2 and two of the vocal learning measures, vocal diversity and vocal plasticity, did trend in the directions that would be predicted based on FoxP2’s role in facilitating vocal learning. Future work investigating other neural mechanisms, beyond FoxP2, that may underly persistent vocal learning ability in open-ended learners would be valuable, especially given that numerous vocal learning candidate genes have been identified within Area X in songbirds, the striatal subregion analogous to MMSt in parrots [40]. Given that species with vocal learning share similar underlying neuroarchitecture and neurogenetic mechanisms for this trait, further investigation in other open-ended vocal learners, such as humans, would be valuable in better understanding the mechanisms that support persistent learning ability throughout the adult lifespan.

## Conclusions

Assessing both neural expression of FoxP2 in the parrot vocal learning nucleus, MMSt, and contact call learning in naturalistic flocks of captive budgerigars of two different adult age classes, we find support for continued vocal learning into late adulthood with largely the same fidelity as in 1st year adults, with respect to vocal plasticity and vocal convergence to flockmates. Although older adults exhibited higher FoxP2 expression in MMSt compared to younger adults, they maintained a similarly low MMSt/VSP expression ratio characteristic of persistent vocal learning. This is the first experimental study to confirm that persistent downregulation of FoxP2 in the budgerigar MMSt, previously hypothesized to be a key contributing factor in the apparent life-long learning ability of this parrot, is maintained throughout later adulthood.

## Data Availability

All data and code generated in this study have been deposited at Dryad and are publicly available as of the date of publication (DOI: 10.5061/dryad.gb5mkkwvj).
